# Factors associated with the subspecialty choices of internal medicine residents in Canada

**DOI:** 10.1186/1472-6920-8-37

**Published:** 2008-06-26

**Authors:** Leora Horn, Katina Tzanetos, Kevin Thorpe, Sharon E Straus

**Affiliations:** 1 Department of Medical Oncology, Vanderbilt University, 777 Preston Research Building, 2220 Pierce Avenue, Nashville, TN 37232, USA; 2 Department of Medicine, University of Toronto, 200 Elizabeth Street, Toronto General Hospital, 14 EN, Room 224, Toronto, ON, M5G 2C4, Canada; 3 Keenan Research Center in the Li Ka Shing Knowledge Institute, 30 Bond Street, Toronto, ON, M5B 1W8, Canada and Department of Public Health Sciences, Faculty of Medicine, University of Toronto, 155 College Street, 6 Floor, Toronto, ON, M5T 3M7, Canada; 4 Department of Medicine, University of Calgary, Room 1103 South Tower, 1403 29^th ^Street NW, Calgary, AB, T2N 2T9, Canada

## Abstract

**Background:**

Currently, there are more residents enrolled in cardiology training programs in Canada than in immunology, pharmacology, rheumatology, infectious diseases, geriatrics and endocrinology combined. There is no published data regarding the proportion of Canadian internal medicine residents applying to the various subspecialties, or the factors that residents consider important when deciding which subspecialty to pursue. To address the concern about physician imbalances in internal medicine subspecialties, we need to examine the factors that motivate residents when making career decisions.

**Methods:**

In this two-phase study, Canadian internal medicine residents participating in the post graduate year 4 (PGY4) subspecialty match were invited to participate in a web-based survey and focus group discussions. The focus group discussions were based on issues identified from the survey results. Analysis of focus group transcripts grew on grounded theory.

**Results:**

110 PGY3 residents participating in the PGY4 subspecialty match from 10 participating Canadian universities participated in the web-based survey (54% response rate). 22 residents from 3 different training programs participated in 4 focus groups held across Canada. Our study found that residents are choosing careers that provide intellectual stimulation, are consistent with their personality, and that provide a challenge in diagnosis. From our focus group discussions it appears that lifestyle, role models, mentorship and the experience of the resident with the specialty appear to be equally important in career decisions. Males are more likely to choose procedure based specialties and are more concerned with the reputation of the specialty as well as the anticipated salary. In contrast, residents choosing non-procedure based specialties are more concerned with issues related to lifestyle, including work-related stress, work hours and time for leisure as well as the patient populations they are treating.

**Conclusion:**

This study suggests that internal medicine trainees, and particularly males, are increasingly choosing procedure-based specialties while non-procedure based specialties, and in particular general internal medicine, are losing appeal. We need to implement strategies to ensure positive rotation experiences, exposure to role models, improved lifestyle and job satisfaction as well as payment schedules that are equitable between disciplines in order to attract residents to less popular career choices.

## Background

In Canada, there is a growing concern over the imbalance in the numbers and composition of internal medicine subspecialty training programs. Currently, there are more residents enrolled in cardiology training programs than in immunology, pharmacology, rheumatology, infectious diseases, geriatrics and endocrinology combined [1]. Studies on physician resources have predicted critical shortages of generalists within the next 5 years [2]. Similarly, the Canadian Rheumatology Association estimates that by 2026 there will be a 64% shortfall in the number of rheumatologists available to service the population [3]. Other clinical disciplines such as geriatric medicine project similar shortfalls.

The concern about predicted physician shortages in various subspecialties has led medical educators to question the factors that motivate internal medicine residents when making career decisions. Currently, there is no published data regarding the proportion of Canadian internal medicine residents applying to the various subspecialties, or the factors that residents consider important when deciding which subspecialty to pursue. Research to date extrapolates from data focusing on medical students to determine what factors influence residents' career decisions. The few studies that are available to inform this area target American trainees, and largely ignore Canadian residents. Financial considerations such as escalating debt and lower future income potential have been identified as a reason why American graduates do not apply to primary practice or consider academic careers [4-8]. In order to ensure a spectrum of physicians that can meet the health care needs of Canadians, we need to ensure continued interest in general internal medicine, as well as some of the other less well-represented sub-specialty practices.

The aim of this study was to determine the distribution of residents entering various sub-specialty programs across Canada and to establish what factors residents consider to be important in determining their career choice. Such information has implications for medical education, policy, and curricular reform. If we are able to identify the variables that attract or dissuade residents from particular subspecialties, medical educators and policymakers could use this information to determine what barriers must be addressed to ensure that all specialties are well-represented to residents making their career choices.

## Methods

In their third year of post-graduate training, Canadian internal medicine residents must decide if they are going to sub-specialize (two to three additional years of training) or continue training in general internal medicine (one additional year of training). Those residents choosing to sub-specialize receive a license in both general internal medicine and the subspecialty they pursue. This two-part study involved both a survey and focus groups on three different cohorts of Canadian residents participating in the post-graduate year 4 (PGY4) internal medicine subspecialty match.

### Part 1: Survey

Survey items were generated following an extensive literature review that produced a list of factors influencing career choices of medical students, residents and physicians [4-18]. Based on this review, the research team, consisting of a geriatrician, general internist and internal medicine resident, generated a list of potentially important variables to resident career decision-making. Using a modified Delphi technique, the team reduced this list to generate a final group of questionnaire items that were included in the survey along with demographic data. There were a total of 24 demographic and 50 non-demographic variables. Prior to administration, the survey was pilot tested by a random sample of five PGY4 residents to ensure the instrument was coherent and had appropriate face validity.

All internal medicine program directors, at the 12 English-speaking Universities across Canada were contacted and invited to collaborate. Residents participating in the PGY-4 internal medicine subspecialty match were contacted via email through their program director and asked to complete a confidential web-based survey. Reminder e-mails were sent to all residents at two and four weeks following the initial email. This strategy of recruitment has been shown to significantly increase survey response rates [19]. Consent was obtained when the resident logged onto the website to complete the survey. The completion of the survey was anonymous. Data was collected via an internet-based system that allowed for automatic data entry into the database.

For purpose of analysis, sub-specialties were grouped as follows: Group 1: procedure based specialties (cardiology, respirology, intensive care and gastroenterology), Group 2: non-procedure based specialties (medical oncology, hematology, infectious disease and nephrology) and Group 3: non-procedure based specialties with declining applicants in the last five years (rheumatology, endocrinology, geriatrics and general internal medicine). Categorical demographic variables were summarized as counts and percentages and number demographic variables were summarized by medians and quartiles. A Pearson's chi-square test was used to test for differences across the three groups for the categorical variable and the Kruskal-Wallis test was used for the numeric variables. The Likert scores of each non-demographic variable were averaged in each group to determine the five highest and lowest ranked variables irrespective of differences among groups. The non-demographic survey items were also analyzed using chi-square test and a false discovery rate controlling procedure was used to adjust for multiple comparisons when determining statistically significant factors [20]. All statistical analyses were completed using *R: A language and environment for statistical computing*. R is an open-source dialect of the S language (S was developed by AT&T) that is maintained by a core team.

### Part 2: Focus group exploration of factors influencing career choice

Four focus groups were conducted in 3 Canadian cities – one each in Vancouver and Halifax and two in Toronto. These sites represent three programs of different size and structure that had participated in our web-based survey. Third year (PGY3) internal medicine residents were invited to participate via e-mail through their program director's office after they had been accepted into their subspecialty training programs. Focus group questions were open ended and based on the results from the previously distributed survey. Participants were encouraged to speak freely and to support their responses with examples. Each focus group lasted 60–120 minutes. Residents signed consent forms prior to beginning the interview, and were given remuneration for their time. All focus group discussions were audio taped and transcribed with identifying information removed. Two study investigators independently analyzed the transcripts drawing on grounded theory and beginning with the first focus group. The comments were separated into categories with thematic labels based on actual words used by participants. A third investigator subsequently reviewed these memos and the transcripts to determine if any additional themes were present. We noted a saturation of themes by the fourth focus group and it was decided not to perform additional focus groups [21,22].

## Results

### Part 1: Survey Data

10 of 12 eligible Universities agreed to participate in this study. 110 PGY3 residents across Canada responded to the survey (54% response rate).

### Demographic Factors

Gender was a significant determinant of specialty choice with 78% of positions in procedure based specialties occupied by men compared with only 39% of positions in non-procedure based specialties with declining interest *X*^2 ^(2, N = 110) = 10, p = 0.006. Residents choosing procedure based specialties and popular non-procedure based specialties were more likely to have applied to multiple programs (p < 0.0001) and to have been involved in research within their specialty during their training. Regardless of the specialty chosen, a significant number of residents developed an interest in their future specialty during medical school (38% – 41%), but their final decision to apply occurred during residency training (73 – 89%) (Table 1).

**Table 1:** Demographics of survey group participants

	Group 1* (N = 49)	Group 2^¶ ^(N = 38)	Group 3^§ ^(N = 23)	P Value
Age	29 (28–30)	28 (27–30)	29 (28–30)	0.2^K^
Male	38 (78%)	23 (61%)	9 (39%)	0.006^X^
# Programs Applied to	5	4	1	<0.001^K^
Graduate degree prior to medical school	14%	11%	21%	0.3^X^
Degree in same field	29%	50%	20%	0.6^X^
Research in field during residency	80%	76%	52%	0.04^X^
Interest in specialty	14%	29%	9%	0.006^X^
Prior to medical school	38%	42%	43%	
During medical school	47%	29%	48%	
During residency				
Decision on specialty	4%	3%	4%	0.6^X^
Prior to medical school	22%	8%	15%	
During medical school	73%	89%	81%	
During residency				
Exposed to subspecialty attending physician on CTU during medical school	40%	30%	50%	0.5^K^
Exposed to subspecialty attending physicians on GIM CTU during residency	50%	22%	44%	0.04^K^

*Group 1: procedural specialties; ^¶^Group 2: non-procedural specialties; ^§^Group 3 : non-procedural specialties with declining interest.^X ^P value determined using chi-square test; ^K ^P valued determined using kruskal walis test

The residents' level of debt, hometown population, percentage of specialists and generalists exposed to during medical school and residency, and prior degrees were not found to be significant factors in career choices. The current university and specialties availability at the university did not affect career choice nor were there differences in the reasons for selecting a particular specialty between training programs.

### Non-demographic Factors

Residents were asked to rank the importance of factors for choosing their specialty on a likert scale (1–5, low – high). There were 2 variables among the 5 most important factors that were common to all three resident groups regardless of future specialty: *intellectual stimulation *and *diversity of clinical spectrum*. Residents choosing procedure and non-procedure based specialties ranked *satisfaction among staff physicians and challenge of diagnostic problems *as important, while their fifth most important variable differed in that *consistent with personality *was important to residents choosing non-procedure based specialties and *ability to do procedures *was important to residents choosing procedure based specialties. Residents choosing non-procedure based specialties with declining interest cited *consistent with personality, lifestyle *and *predictable working hours as a staff *to complete their top 5 (Table 2).

**Table 2:** The five most important factors to residents in making career decisions

	Group 1* (N = 49) Mean (SD)	Group 2^¶ ^(N = 38) Mean (SD)	Group 3^§ ^(N = 23) Mean (SD)
**Consistent with personality**	3.61 (1.11)	**3.97 (0.91)**	**4.26 (0.62)**
Intellectual stimulation	**4.08 (0.81)**	**4.42 (0.79)**	**4.09 (0.73)**
Diversity of clinical spectrum	**4.06 (0.96)**	**4.24 (0.85)**	**3.87 (0.97)**
Challenge of diagnostic problems	**3.86 (0.96)**	**4.03 (0.97)**	3.78 (1.00)
Opportunity to do procedures	**4.14 (0.94)**	2.37 (1.30)	2.57 (1.16)
Satisfaction among staff physicians	**3.69 (0.87)**	**3.84 (0.86)**	3.74 (0.96)
Predictable working hours as a staff	3.00 (1.08)	3.45 (1.01)	**3.83 (0.78)**
Time for leisure as a staff	3.22 (1.03)	3.47 (1.01)	**4.17 (0.72)**

**Bolded values refer to the 5 most important factors to trainees**. *Group 1: procedural specialties; ^¶^Group 2: non-procedural specialties; ^§^Group 3 : non-procedural specialties with declining interest.

The *opportunity to do procedures *(P < 0.001) and the residents *perceived reputation of the specialty among the general population *(P < 0.001) were significantly more important to residents choosing procedure based specialties. Factors that trended towards significance related to lifestyle included *stress among staff physicians*, *time for leisure as a staff *and *work hours during training*. These factors were most important to residents choosing non-procedure based specialties with declining interest. *Work hours as a staff *were also important to residents choosing non-procedure based specialties. *Anticipated salary as a staff *and the *opportunity to provide acute/inpatient care *was most important to procedure based specialty residents. The *opportunity to provide continuity of care *and *to deal with chronic illnesses *were cited as important to residents choosing non-procedure based specialties and non-procedure based specialties with declining interest. Residents selecting non-procedure-based specialties were also more concerned with the *patient population treated *and *a need to contribute towards *society.

### Part 2: Focus Groups

Twenty-two residents participated in our focus group discussions (5–6 residents per focus group). The demographics of the focus groups are presented in Table 3. Four major themes were identified; 1) mentorship, role models and experience on a rotation, 2) patients, practice type and personal fit 3) lifestyle and family 4) future job opportunities and finances.

**Table 3:** Demographics of focus group participants

	Group 1* (n = 10)	Group 2^¶ ^(n = 6)	Group 3^§ ^(n = 6)
Age	29.8	30.3	29.2
Male (%)	60%	33.3%	50%
Married (%)	50%	50%	66.7%
Toronto* (11)	45.5%	27.3%	27.3%
Vancouver (5)	80.0%	20.0%	0
Halifax (6)	16.7%	33.3%	50%

*Group 1: procedural specialties; ^¶^Group 2: non-procedural specialties; ^§^Group 3: non-procedural specialties with declining interest

### Mentorship, role models and experience on a rotation

Residents identified instances where they encountered both positive and negative role models that may have influenced their career decisions. Lack of exposure to fellows may have negatively impacted a residents' decision to apply to a specialty. This was highlighted in a statement by a participant:

"I wonder whether a factor was the amount of exposure you get to fellows. Like in geriatrics I've never seen a fellow and on general medicine you barely saw a fellow. It's mostly staff."

Effective mentorship relationships were more likely to develop informally with either staff or fellows. At one institution where a formal, assigned mentorship program was available to residents, the residents did not perceive this as a helpful initiative and suggested more guidance was needed around the development of a mentoring relationship as outlined by the following statement:

*"I don't know how good those forced [mentorship] programs really are.. it's a personal process*.

Female residents were more likely to look to female mentors that they could emulate both at work and home. One participant stated:

"A lot of the mentors that I think about are the ones that were actual females with kids that had them during their residency that I've met along the way and they were a great resource."

### Patients, practice type and personal fit

Residents were dissuaded from specialties where they felt fatigued, overworked and not confident. They chose specialties where they were stimulated and enjoyed the type of work they were doing as highlighted by the response from one resident:

"you're just very happy to be at work every day and wonderful things with lungs, outpatients, older patients, really sick patients, ICU procedures, bronching .... I haven't been on any other rotation that I thought like I could do this every day and get excited every day."

Participants reported positive experiences on general medicine in non-university affiliated centers where staff appeared to enjoy their jobs more than in the academic environment. The residents noted that subspecialty staff in university-affiliated centers also appeared to enjoy their jobs more than general internists as suggested by one participant:

"I had mentors in GIM but I didn't choose it because I don't think they are as happy in general. Like their satisfaction isn't as good as the ones that are in specialties."

### Lifestyle and Family

Lifestyle was an important factor to most participants. Residents were looking for flexibility in their careers and opportunities to do things outside of the hospital and clinic with one resident supporting their career choice by stating:

"It means not having to spend all of my time at the hospital. Opportunities to do other things besides medicine."

Participants realized that in choosing certain specialties, such as cardiology, they would have to make sacrifices in lifestyle, as noted by one resident who said:

"Lifestyle played a big role and that was one of the reasons I eliminated ICU and cardiology. I didn't like the prospect of doing in-house call during fellowship..."

### Financial and Job Opportunities

Although residents do not have specific salary expectations, finances seemed to be an important consideration. As stated by one resident entering a procedure based specialty,

"I went through all this school and I'm not going to choose a specialty where I make$150-$200 000. I want a higher earning than that."

In contrast, residents choosing non-procedure based specialties with declining interests believed that salary is less important than job satisfaction as supported by the following statement from a resident:

"In choosing rheumatology money was very, very low down on the list as a defining characteristic."

Residents perceived that an academic career would result in less earning potential than a community career and they believed that this is particularly true in geriatric and general internal medicine with one resident stating:

"I don't want to work a lot harder [in an academic institution] to generate the same salary [as a community cardiologist]."

Finally, participants recognized that it is easier to get a job in specialties with declining interest such as geriatrics. In choosing their subspecialty, residents attempt to assess future job opportunities and the ability to combine research with clinical work. This was highlighted in the statement from a participant:

"The chances of being able to walk into a position [as an academic cardiologist] is actually quite low and so that's very negative."

## Discussion

From 1998 to 2003 there has been a progressive increase, from 28.3% to 43.1%, in the number of residents completing their training in procedure based specialties. At the same time, there has been a decrease, from 47.5% to 31.2%, in trainees completing training in non-procedure based specialties with declining interest[1] Although the percentage of females in medicine continues to increase and from our study it appears that females are more likely to occupy positions in specialties such as geriatrics, general internal medicine, rheumatology and endocrinology, this does not translate into an increase in the number of these specialists occupying the Canadian work force. This situation is likely because this group only represents 20% of all residents entering their PGY4 year, less than half of the number of residents entering procedure-based specialties.

Mentorship impacts on career development and productivity [23-26]. The University of Toronto and other institutions have implemented formal, assigned mentorship programs in an attempt to improve resident-faculty interactions. However, residents in this study found that these programs were not helpful, and they preferred to choose a mentor from faculty they encountered during clinical rotations. Therefore, faculty in specialties with declining interest could attempt to ensure they are visible, available and approachable to residents seeking guidance with their careers. Similar to a recent study that identified residents as influencing students' career choices[27], this study recognized fellows as a source of mentorship and role modeling. Participants also recognized the lack of exposure to fellows in general internal medicine and geriatrics during training. Ensuring adequate exposure to fellows on general medicine rotations may improve the perception of and applications to a subspecialty. However this may prove difficult given the scarcity of general medicine and geriatric medicine fellows in Canada. Similar to previous studies involving surgical residents [28] and medical students [29], we found gender to be an important factor in career decisions. Female residents identified a female role model as important in aiding them in their subspecialty choices. Female residents are more likely to consider a subspecialty where they see successful female faculty members[28].

Traditionally, an emphasis has been placed on medical students being exposed to and having positive experiences during their general medicine rotation in order to increase applications [9,12,13,15]. We have observed that a residents' experience on a rotation appears to be equally important in influencing their subspecialty choices. Several residents mentioned their experience on general medicine in a non-academic centre was extremely positive but their exposure occurred too late in their training to influence their career decisions. However, some programs, such as the University of British Columbia, have mandatory non-academic general medicine rotations early in residency training and this does not appear to have resulted in an increased number of applicants to general medicine. In addition, specialties such as cardiology and critical care can be as demanding as general internal medicine but do not show the same decline in applicants.

Our study confirms previous observations that residents are placing greater influence on lifestyle in choosing their subspecialty careers [11,18,30]. One study found that lifestyle was the most common reason cited for general surgical residents leaving their programs [31]. Lifestyle of a staff physician appears to be a very important variable that incorporates many aspects including predictable work hours, stress, on-call hours and salary. These factors were all considered important to residents choosing non-procedural based careers.

Studies of residents in the United States, have indicated that financial considerations make the greater compensation in procedure orientated specialties appealing [32,33]. We also found that finances were important to residents, especially those choosing procedural based specialties. A study of Canadian residents found that they carry high credit-card and educational debts and that they anticipate substantial earnings and postpone saving to repay debts and finance retirement consumption to a period later in life [34]. Level of debt has been shown to increase resident stress and their likelihood of considering income potential when choosing a specialty [35]. The Canadian Society of Internal Medicine (CSIM) recently cited long standing inequalities in remuneration between procedural and cognitive based specialties as a barrier in resource planning [2,36]. Several authors have highlighted a need to reform policies to reduce the inequalities in reimbursement between procedure-related and patient-centered practices in order to renew interest in general internal medicine [36,37]. However, some institutions have implemented salaried positions for geriatric medicine, and this has not led to an increased interest in geriatrics. Therefore such a simple solution may not be adequate for increasing the number of residents applying to and entering what are currently considered lower paid specialties.

This study is limited in that the survey was performed after trainees had matched to their sub-specialty program. However, more than 90% of trainees are accepted to their first choice of specialty and therefore their answers represent motives for their choice not justification. A total of 136 trainees participated in the study from three different cohorts; two cohorts of trainees entering their subspecialty programs in July 2004 (University of Toronto only) and 2005 (all Canadian trainees), representing 110 trainees. There was no difference in responses for subspecialty groups between program locations or years. In addition themes that emerged during focus group discussions (an additional 22 trainees entering subspecialty training in 2006) validated findings that emerged from the survey data suggesting these results can be generalized to all trainees in Canada.

The question remains as to how we can increase the appeal of the non-procedural based specialties with declining interest in order to ensure a more equitable distribution of the physician workforce. There are some factors that are inherent to the individual disciplines that cannot be changed, such as the fact that procedural based specialties are exactly that, procedural based. In Canada the general internist provides consultative support to general practitioners while occasionally requiring additional services from sub-specialists, chiefly when high-technology procedural care is required [38]. There are a growing number of communities that require generalists with expertise in procedural skills. Training programs have a responsibility to provide generalists with options for developing their procedural skills [39]. Training generalists to perform procedures is one way of responding to the needs of the Canadian population by potentially increasing the number of residents entering generalists practice as well as ensuring access to certain procedures for patients. Most likely a multi-factorial solution is required to meet the challenge of addressing imbalances including enhanced mentorship opportunities, development of effective mentorship initiatives and innovative funding strategies.

The decline in generalists and other cognitive specialties has been a gradual process that needs to be reversed in order to sustain the Canadian health care system. Unfortunately, residents may not appreciate the impact of this within the Canadian health care system. Residents who participated in our study believed that "specialists or family physicians will take on more general medicine practices". However, the general internist has a unique acumen that cannot be practiced part time or without sufficient training. There is also a role for program directors, academic generalists and policy makers to implement strategies to improve the image of general medicine by improving morale on clinical teaching units, improving access to general internists for residents in training and increasing compensation for practicing physicians in an effort to increase interest and applicants to general medicine programs in the future.

## Conclusion

This study suggests that internal medicine trainees, and particularly males, are increasingly choosing procedure-based specialties while non-procedure based specialties, and in particular general internal medicine, are losing appeal. Mentorship, specifically from faculty of the same gender for females, exposure to fellows and a residents experience on a rotation, lifestyle and finances all appear to be important factors to Canadian residents when making their subspecialty selections. We need to implement strategies to ensure positive rotation experiences, exposure to role models, improved lifestyle and job satisfaction as well as payment schedules that are equitable between disciplines in order to attract residents to less popular career choices.

## Competing interests

The authors declare that they have no competing interests.

## Authors' contributions

LH, KT and SS conceived of the study, participated in its design and coordination. KT performed the statistical analysis. All authors read and approved the final manuscript.

## Pre-publication history

The pre-publication history for this paper can be accessed here:


